# Stable isotope informed genome-resolved metagenomics reveals that Saccharibacteria utilize microbially-processed plant-derived carbon

**DOI:** 10.1186/s40168-018-0499-z

**Published:** 2018-07-03

**Authors:** Evan P. Starr, Shengjing Shi, Steven J. Blazewicz, Alexander J. Probst, Donald J. Herman, Mary K. Firestone, Jillian F. Banfield

**Affiliations:** 1 Department of Plant and Microbial Biology, University of California, Berkeley, CA 94720 USA; 2 Lincoln Science Centre, AgResearch Ltd, Christchurch, 8140 New Zealand; 3 Nuclear and Chemical Sciences Division, Lawrence Livermore National Laboratory, Livermore, 94550 USA; 4 Biofilm Center, University of Duisburg-Essen, 45141 Essen, Germany; 5 Department of Environmental Science, Policy, and Management, University of California, Berkeley, CA 94720 USA; 6 Earth Sciences Division, Lawrence Berkeley National Laboratory, Berkeley, California 94704 USA; 7 Department of Earth and Planetary Science, University of California Berkeley, Berkeley, CA 94720 USA

**Keywords:** Stable isotope probing, Complete genome, Saccharibacteria, Metagenomics, Rhizosphere

## Abstract

**Background:**

The transformation of plant photosynthate into soil organic carbon and its recycling to CO_2_ by soil microorganisms is one of the central components of the terrestrial carbon cycle. There are currently large knowledge gaps related to which soil-associated microorganisms take up plant carbon in the rhizosphere and the fate of that carbon.

**Results:**

We conducted an experiment in which common wild oats (*Avena fatua)* were grown in a ^13^CO_2_ atmosphere and the rhizosphere and non-rhizosphere soil was sampled for genomic analyses. Density gradient centrifugation of DNA extracted from soil samples enabled distinction of microbes that did and did not incorporate the ^13^C into their DNA. A 1.45-Mbp genome of a Saccharibacteria (TM7) was identified and, despite the microbial complexity of rhizosphere soil, curated to completion. The genome lacks many biosynthetic pathways, including genes required to synthesize DNA de novo. Rather, it requires externally derived nucleotides for DNA and RNA synthesis. Given this, we conclude that rhizosphere-associated Saccharibacteria recycle DNA from bacteria that live off plant exudates and/or phage that acquired ^13^C because they preyed upon these bacteria and/or directly from the labeled plant DNA. Isotopic labeling indicates that the population was replicating during the 6-week period of plant growth. Interestingly, the genome is ~ 30% larger than other complete Saccharibacteria genomes from non-soil environments, largely due to more genes for complex carbon utilization and amino acid metabolism. Given the ability to degrade cellulose, hemicellulose, pectin, starch, and 1,3-β-glucan, we predict that this Saccharibacteria generates energy by fermentation of soil necromass and plant root exudates to acetate and lactate. The genome also encodes a linear electron transport chain featuring a terminal oxidase, suggesting that this Saccharibacteria may respire aerobically. The genome encodes a hydrolase that could breakdown salicylic acid, a plant defense signaling molecule, and genes to interconvert a variety of isoprenoids, including the plant hormone zeatin.

**Conclusions:**

Rhizosphere Saccharibacteria likely depend on other bacteria for basic cellular building blocks. We propose that isotopically labeled CO_2_ is incorporated into plant-derived carbon and then into the DNA of rhizosphere organisms capable of nucleotide synthesis, and the nucleotides are recycled into Saccharibacterial genomes.

**Electronic supplementary material:**

The online version of this article (10.1186/s40168-018-0499-z) contains supplementary material, which is available to authorized users.

## Background

The candidate phyla radiation (CPR) comprises a large fraction of the bacterial domain [1, 2]. Within the CPR, Saccharibacteria, formerly TM7, is one of the most captivating phyla because of the wide diversity of habitats in which it is found, including activated sludge, human and dolphin oral cavities, seawater, aquifer sediment, soil, and cockroach guts [3–10]. Much has been learned about the metabolism of Saccharibacteria and their influence on their environment, for instance, the possible immunosuppressive capabilities of an episymbiont of *Actinomyces odontolyticus* in human mouths and bulking issues caused in wastewater treatment plants [7, 11]. There have been previously proposed subdivisions within the Saccharibacteria phylum, but currently the majority of our genomic data comes from organisms which fall within Subdivision 3 and there exists very little genomic representation of Subdivision 1 [4, 12]. Organisms in Subdivision 1 are predominantly found in environmental samples [13]. One of the reasons why there may be less genomic data available for Saccharibacteria in Subdivision 1 is because of the high microbial diversity of environmental samples, especially soil, which makes sequencing efforts more challenging. Based on 16S ribosomal RNA gene surveys, we know that Saccharibacteria occur in the rhizosphere, but little is known about their metabolism and how they differ from related organisms growing in other environments [14, 15].

There are six previously reported, closed, and circularized genomes for Saccharibacteria (one from a wastewater treatment plant [16], human mouth [7], two from sediment [1, 8], and two from a thiocyanate remediation wastewater reactor [17]) and some partial genomes. Based on genomic analyses, it was previously predicted that Saccharibacteria are anaerobic fermenters, despite the documentation of one genome encoding a single-subunit of NADH: ubiquinone dehydrogenase and a complete ubiquinol oxidase, which was explained as an oxygen-scavenging mechanism [8]. It has also been documented that Saccharibacteria are able to grow in the presence of oxygen [3, 7, 18]. Previous soil SIP work has shown that Saccharibacteria DNA became labeled in the presence of ^13^C cellulose and toluene [19, 20]. Labeling experiments have indicated that ^13^CO_2_ can be traced from plant fixation into the DNA of rhizosphere-growing microbes [21, 22]. Here, we found that a Saccharibacteria population in rhizosphere soil incorporated ^13^C into its genomic DNA, indicating that this population was active and dividing. We report the complete genome for this organism and show how analysis of its metabolism sheds light on the pathway by which the label was incorporated. We compare the genome to those of other complete Saccharibacteria to address the question of how this bacterium is adapted to live in soil and specifically in the rhizosphere.

## Results

**Fig. 1:**
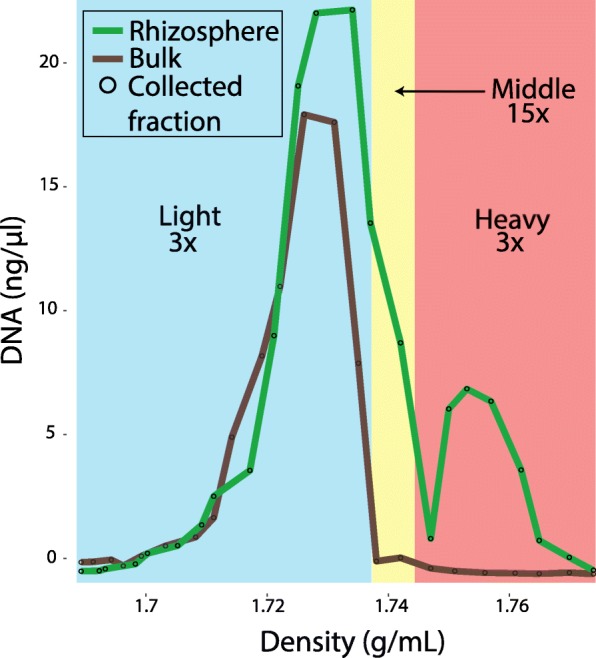
Stable isotope fraction determination. This plot shows the distribution of densities and concentrations of DNA extracted from week 6 rhizosphere and bulk soil following density centrifugation. The black circles on the curves represent individual fraction measurements. The three fractions are designated as light (blue shading), middle (yellow shading), and heavy (red shading). The top numbers indicate the normalized coverage of the *T*. *rhizospherense* genome in each fraction. The *T*. *rhizospherense* genome had < 1× coverage in each bulk soil fraction

After 6 weeks in labeling chambers, *Avena fatua* shoots were highly labeled (~ 94 atom% ^13^C). DNA was extracted from the rhizosphere and bulk soil samples. By comparing the density separation of the rhizosphere community DNA to the bulk community DNA, we were able to define un-enriched (light), partially ^13^C-enriched (middle), and highly ^13^C-enriched (heavy) fractions (Fig. 1). Based on the cutoff values for these fractions, 32 density-separated fractions for the rhizosphere sample were then combined, generating light, middle, and heavy fractions (the bulk sample only contains light and middle fractions due to the absence of ^13^C-enriched DNA) (Fig. 1).

The light, middle, and heavy density separated fractions from the rhizosphere and bulk samples were sequenced and subjected to genome-resolved metagenomic analyses (Additional file 1: Table S1). From the rhizosphere middle fraction, we assembled 210 Mbp of scaffolds larger than 1 kbp. One especially large scaffold was assembled de novo and could be circularized. Local assembly errors were identified and corrected and three scaffolding gaps were filled by manual curation. Manual curation made use of unplaced paired reads that were mapped back to the gap boundaries to fill gaps. The complete, closed genome is 1.45 Mb in length with a GC content of 49.95%. We were able to recover a single chromosome and we detected no integrated phage or plasmids. The genome was most abundant in the rhizosphere middle fraction at 15× coverage, but was also present in the rhizosphere heavy and light fractions at ~ 3× normalized coverage. The genome had less than 1× coverage in the non-rhizosphere soil (Additional file 1: Table S1).

DNA buoyant density in a cesium chloride solution is a function of both the extent of isotopic enrichment and the GC content. Low GC has a lower buoyant density as compared to higher GC DNA [23]. The completed, closed genome has a lower GC content (49.95%) than the rest of the rhizosphere middle fraction assembly (average GC content of scaffolds larger than 1000 bp is 66%), which indicates ^13^C may have been incorporated into the DNA. The rhizosphere middle fraction where the genome was mainly detected had a density of 1.737–1.747 g/ml. Given that natural abundance DNA with 49.95% GC content would have a density of ~ 1.71 g/ml [24], we estimate that the DNA from which the genome was assembled was at least 50% enriched in ^13^C.

**Fig. 2:**
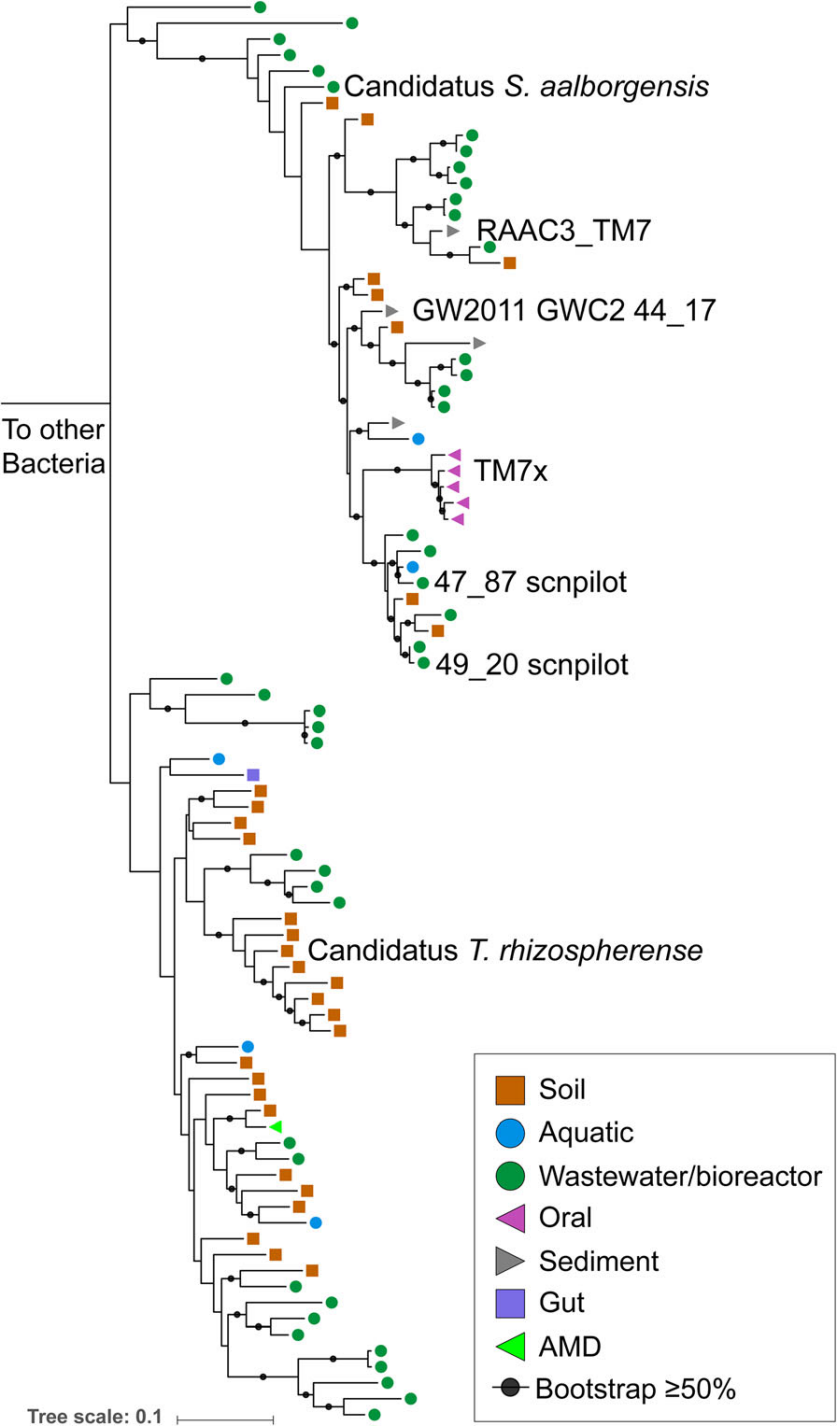
Phylogeny of Saccharibacteria based on 16S rRNA gene sequences. The maximum-likelihood tree shown was constructed from an alignment containing representative Saccharibacteria. Symbols indicate the environmental origin of the NCBI sequence. Named branches indicate the complete genomes included in this study. The tree scale bar indicates nucleotide substitutions per site. Bootstrap values ≥ 50% are indicated by black dots

The genome has 1531 protein coding sequences (Additional file 2: Table S2) and a full complement of tRNAs (46 in total). The 5S rRNA, 23S rRNA, and 16S rRNA genes are in a single locus that also includes Ala and Ile tRNA genes. Based on the sequence of the 16S rRNA gene, the genome was assigned to be a member of the Saccharibacteria phylum. The closest 16S rRNA gene sequences in NCBI are from the rhizosphere of *Pinus massoniana* (Fig. 2) [25]. The most closely related genomically described organism is Candidatus *Saccharimonas aalborgensis* from activated sludge with 84% identity across the full-length 16S rRNA gene [16]. We propose the name *Candidatus* “Teamsevenus rhizospherense” for the organism described here, given the derivation of the genome from the rhizosphere. In accordance with the phylogenetic analysis, we renamed Subdivision 1 to *Candidatus* Soliteamseven because this genome is the first described Candidatus species of this clade. The representatives of this clade are mostly found in soil, therefore, we propose the complete taxonomic descriptor: Phylum: *Candidatus* Saccharibacteria, Class: *Candidatus* Soliteamseven, Order: *Candidatus* Teamsevenales, Family: *Candidatus* Teamsevenaceae, Genus: *Candidatus* Teamsevenus, Species: *Candidatus* rhizospherense.

We calculated the GC skew and cumulative GC skew across the closed *T*. *rhizospherense* genome and found the symmetrical pattern typical for bacteria, with a single peak and trough indicative of the terminus and origin of replication (Additional file 3: Figure S1). This result both validates the accuracy of the circularized genome and confirms that Saccharibacteria use the typical bacterial pattern of bi-directional replication from a single origin to the terminus (as do some Peregrinibacteria, another group of CPR bacteria [26]). The start of the genome was adjusted to correspond to the predicted origin, which lies between the DNA polymerase III subunit beta and the chromosomal replication initiator protein. It has a full set of ribosomal proteins, except for L30, which is uniformly absent in CPR bacteria [1].

### Biosynthetic pathways

**Fig. 3:**
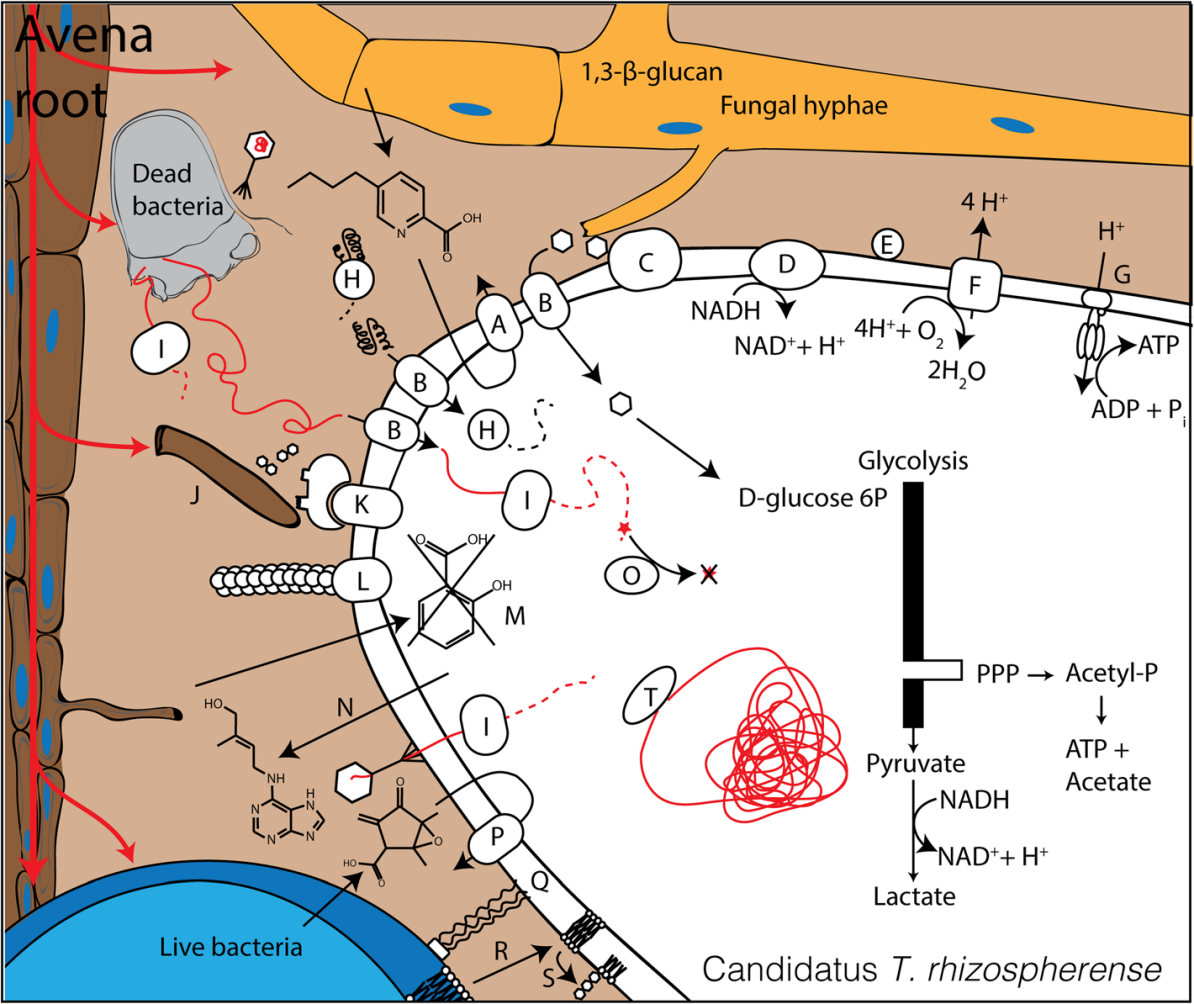
Cell diagram of *T*. *rhizospherense*. (A) fusaric acid resistance machinery, (B) unidentified importer, (C) glucan 1,3-beta-glucosidase, (D) NADH dehydrogenase II, (E) blue-copper protein, (F) cytochrome bo_3_ ubiquinol terminal oxidase, (G) F-type H+-transporting ATPase, (H) peptidase, (I) nuclease, (J) root hair, (K) cellulosome, (L) type IV pilus, (M) salicylate hydroxylase, (N) zeatin production, (O) removal of oxidized nucleotides, (P) various antibiotic resistance mechanisms, (Q) intercellular attachment, (R) scavenging of lipids, (S) production of phosphatidyl myo-inositol mannosides, (T) DNA repair machinery

The *T*. *rhizospherense* genome encodes a number of enzymes for the conversion of nucleotides to NMP, NDP, and NTP and formation of RNA. In addition, we identified genes to phosphorylate G, C, and U. However, the organism lacks the genes required to synthesize 5-phospho-alpha-d-ribose-1-diphosphate (PRPP). Further, it lacks essentially all of the steps for synthesis of nucleotide bases and the pathways that would convert PRPP to inosine monophosphate or uridine monophosphate. *T*. *rhizospherense* may have a novel nucleotide biosynthesis pathway, but this is unlikely as nucleotide biosynthesis remains highly conserved across domains [27] and the pathway can be recognized in some CPR bacteria [26]. Thus, we infer that *T*. *rhizospherense* did not de novo synthesize its nucleotides but rather acquired them from an external source. The genome encodes several nucleases, an external micrococcal nuclease, and an oligoribonuclease for the breakdown of externally derived DNA and RNA (Fig. 3). The mechanism for DNA and RNA import is unknown, as we did not identify nucleotide transporters. However, there are a number of transporters with unidentified specificity that could be involved in DNA or nucleotide uptake or the type IV pili could be responsible for this function. A large portion of the genome is dedicated to DNA and RNA repair mechanisms. There are 20 8-oxo-dGTP diphosphatase genes that prevent the incorporation of oxidized nucleotides. These enzymes may be required given that the nucleotides may be scavenged from dead cells and could have accumulated extensive DNA damage. Access to damaged DNA may be a consequence of life in a mostly aerobic environment, a seemingly unusual condition for members of the Saccharibacteria phylum. All other complete genomes were found in mostly anaerobic environments and encode fewer genes with this function.

The *T*. *rhizospherense* genome does not encode the ability to synthesize any amino acids de novo from central metabolites. However, it encodes genes to generate amino acids from precursors (e.g., valine and leucine from 2-oxoisovalerate, isoleucine from 2-methyl-2-oxopentanoate, and histidine from l-histidinol phosphate) and to interconvert some amino acids (serine and glycine). We identified genes for proteases that could breakdown externally derived proteins. No amino acid-specific transporters were annotated, but several transporters of unknown function could import the amino acids. There is little evidence to suggest that externally derived amino acids are broken down for use in the TCA (the only TCA cycle gene identified is a fumarate reductase subunit) or other cycles.

*T*. *rhizospherense* appears unable to synthesize fatty acids, yet it encodes three copies of the 3-oxoacyl-(acyl-carrier protein) reductase, five copies of acyl-CoA thioesterase I, and two copies of SGNH hydrolase indicating the capacity for fatty acid hydrolysis and conversion. We found that the genome contains a number of genes for sequential steps in the glycerophospholipid metabolism pathway. *T*. *rhizospherense* may incorporate phosphatidylcholine (possibly derived from eukaryotes), 1,2 diacyl sn-glycerol-3P (from bacteria), or phosphatidylethanolamine (the main bacterial phospholipid) and may be able to interconvert the compounds using a gene annotated as phospholipase D. We identified a putative phosphatidate cytidylyltransferase that could add a head group to 1,2 diacyl sn-glycerol-3P forming CDP-diacylglycerol. This may be able to be converted into three products: phosphatidylglycerophosphate (via CDP-diacylglycerol-glycerol-3-phosphate 3-phosphatidyltransferase), or to cardiolipin (via cardiolipin synthase), or to phosphatidyl-1D-myo-inositol (via CDP-diacylglycerol-inositol 3-phosphatidyltransferase).

Interestingly, phosphatidyl-1D-myo-inositol is the precursor for generation of phosphatidylinositol mannosides, glycolipids that are decorated by a chain of mannose molecules and that are found in the cell walls of *Mycobacterium* [28]. There are several genes for the first step in phosphatidylinositol mannoside biosynthesis, which involves modification of phosphatidyl-1D-myo-inositol by addition of mannose. These include phosphatidylinositol alpha-mannosyltransferase (three copies) and a single copy of alpha-1,6-mannosyltransferase. Subsequently, a polyprenol-P-mannose α-1,2-mannosyltransferase (CAZy glycosyltransferase family 87) adds another mannose group. Other mannose additions may involve the three copies of dolichol-phosphate mannosyltransferase, which transfer mannose from GDP-mannose to dolichol phosphate a mannose carrier involved in glycosylation [29]. Thus, although *T*. *rhizospherense* appears to be unable to synthesize fatty acids, it appears to encode a number of genes that may be involved in the interconversion of membrane lipids, including phosphatidylinositol mannosides, if provided 1,2 diacyl sn-glycerol-3P.

### Central metabolism and energy generation

Interestingly, the *T*. *rhizospherense* genome encodes a simple, two-subunit cellulosome that may be used to attach to and degrade plant or microbially derived cellulose to cellobiose. Cellobiose is likely converted to d-glucose via one of 14 different glycosyl hydrolases. The genome also encodes genes for the production of d-glucose via breakdown of starch/glycogen and trehalose. The genome contains several genes for hydrolysis of 1,3-β-glucan, one of the most common fungal cell wall polysaccharides [30], to d-glucose. A significant portion of plant root exudation are sugars that could be fed directly into the *T*. *rhizospherense* metabolism [31]. d-glucose can be converted to d-glucose-6P and d-fructose-6P and fed into the glycolysis pathway. The genome lacks a key step in the glycolysis pathway, the 6-phosphofructokinase enzyme, which takes fructose-6P to fructose 1,6-bisphosphate. However, the missing step is compensated for by the genes in the pentose phosphate pathway that convert fructose-6P to glyceraldehyde-3P which can then continue in the glycolysis pathway. This is a common workaround strategy in many members of the CPR, which frequently lack 6-phosphofructokinase [32]. The product of the glycolysis pathway with the pentose phosphate pathway workaround is pyruvate. The pyruvate is then likely converted to d-lactate by the predicted d-lactate dehydrogenase protein, which would replenish the NAD^+^ pool under fermentative conditions. Pyruvate appears not to be converted to acetyl-CoA, since the genome lacks pyruvate dehydrogenase and pyruvate ferredoxin oxidoreductase. Acetyl-CoA metabolism also appears to be lacking in the Saccharibacteria represented by the six other publicly available complete genomes.

The *T*. *rhizospherense* genome encodes a xylulose-5-phosphate/fructose-6-phosphate phosphoketolase that may convert d-xylose-5P derived from the pentose phosphate pathway to acetyl-P. The neighboring gene is annotated as an acetate kinase, which may convert acetyl-P to acetate with the production of ATP. Thus, we predict growth via fermentation under anaerobic conditions. ATP also can be produced by a F-type H^+^ transporting ATPase.

In glycolysis, NAD^+^ is consumed to form NADH that can be regenerated via what appears to be a linear electron transport chain that includes a single subunit NADH dehydrogenase (*ndh*). We identified several predicted active site residues expected for function (Additional file 4: Figure S2A). Modeling revealed a close secondary structure match between the *T*. *rhizospherense* protein and the Ndh characterized from *Caldalkalibacillus thermarum* [33]. The large evolutionary distance between *T*. *rhizospherense* and *C*. *thermarum* likely accounts for differences in some active site residues.

The genome encodes a few genes in an incomplete pathway for production of quinone-based molecules, two of which are in multicopy (five copies of genes annotated as *ubiG* and two copies as *ubiE*). We suspect that quinone is scavenged from an external source. We identified genes for a cytochrome bo_3_ ubiquinol terminal oxidase (*cyo*). Subunit I contains some functional residues as well as the residues that distinguish it from cytochrome-c oxidases (Additional file 4: Figure S2B), again this discrepancy in the functional residues may be due to the evolutionary distance between *Escherichia coli* and Saccharibacteria [34]. These genes were also reported in a previous study [8]. The terminal oxidase requires heme to function; however, only the final step of heme biosynthesis is predicted in the genome with a protein annotated as protoheme IX farnesyltransferase. We believe *T*. *rhizospherense* may scavenge heme from the environment as we propose it does for nucleotides, amino acids, and quinones. Five FNR family transcriptional regulators may serve to detect O_2_. If O_2_ is available, it may be possible for electrons to be passed linearly from the Ndh to the cytochrome bo_3_ ubiquinol terminal oxidase, which pumps four protons with the reduction of oxygen [35].

Several predicted proteins such as a blue-copper protein and NADH-quinone oxidoreductase subunit L (*nuoL*)-related protein also may be involved in electron transfer; NuoL is known to contribute to membrane potential in Ndh systems [36]. The *nuoL* gene was found in the same region as the *ndh* and ATPase. Three cytosolic NADPH:quinone oxidoreductase genes were identified. These may reduce semiquinone (SQ) formed under conditions of high O_2_ availability to prevent reaction of SQ with O_2_ to form oxygen radicals [37]. We identified a novel protein that we predict may be involved in production of an electrochemical gradient. It contains two of the domains found in separate subunits of the Na^+^-translocating NADH-quinone reductase (*na(+)-NQR*), contains an FeS cluster domain, and is likely associated with the cytoplasmic membrane. Although speculative, we hypothesize that this protein is a part of the electron transport chain, converting NADH to NAD^+^. One domain of the protein is similar to *na(+)-nqr* subunit F-like domain which may oxidize NADH and transfer electrons to the iron-sulfur domain, and the *na(+)-nqr* subunit B-like domain could form the Na^+^ translocating channel [38].

### Other predicted capacities indicative of lifestyle

The genome lacks a CRISPR-Cas defense system, though one has been noted in the genome of a separate Saccharibacteria [10]. Additionally, we did not find any associated phage or mobile elements, although there were a number of labeled phage contigs in the same sample which will be detailed in a separate publication.

We predict the capacity for twitching motility due to the presence of genes required for type IV pilus assembly and *pilT*, the twitching motility gene. Type IV pili may be involved in DNA uptake or attaching to other cells, root surfaces, or solids. There are several genes for pseudo-pili, and an autotransporter adhesin that may also be involved in cellular attachment. Also, annotated were two CAZy carbohydrate-binding module family 44 genes for binding to cellulose and the capacity for biosynthesis of cellulose that could be used to attach to plant surfaces [39].

We identified genes encoding for laccase and pyranose 2-oxidase, which may be used for lignin breakdown or detoxification of phenolics, and genes for the detoxification of lignin byproducts, including 3-oxoadipate enol-lactonase and 4-carboxymuconolactone decarboxylase [40].

Interestingly, despite the small size of the genome and lack of many core biosynthetic pathways, we identified genes whose roles may be to modulate plant physiology, consistent with a close relationship with the plant. For example, we identified genes for the production of *cis*-zeatin (a plant hormone) from isoprenoid precursors (but a pathway for formation of the precursor isopentenyl-PP was not present, so the precursors are likely scavenged). The genome encodes a protein that appears to be cytokinin riboside 5′-monophosphate phosphoribohydrolase also known as “Lonely Guy,” a cytokinin (a plant growth hormone)-activating enzyme. We also found a gene encoding salicylate hydroxylase, which breaks down salicylic acid, a plant defense signaling molecule.

In addition to genes involved in plant interaction, we predict the capability to interact with other soil microbes. The genome contains *N*-acyl homoserine lactone hydrolase, a gene for quorum quenching of other soil microbes and a gene to form 3′,5′-cyclic-AMP, which may be involved in intracellular signaling. We predict the presence of genes that confer resistance to bacterially produced antibiotics (beta-lactams, streptomycin, oleandomycin, methylenomycin A, vancomycin, and general macrolides) based on sequence similarity. A gene annotated as phosphatidylglycerol lysyltransferase may produce lysylphosphatidylglycerol, a membrane lipid involved in cationic antimicrobial peptide resistance [41]. We also found a gene that may confer resistance to fusaric acid, an antibiotic made by a common fungal pathogen of grass [42]. This fungus was found to be growing in the rhizosphere (data not shown). Interestingly, there is a possible secreted toxin gene that encodes a 2487 amino acid protein; it contains domains found in polymorphic toxins, rearrangement hotspot repeats, YD repeats, a PA14 domain, and a galactose-binding domain and the neighboring gene encodes an immunity protein [43].

### Comparative genomics

The *T*. *rhizospherense* genome is 28% larger than the largest reported complete Saccharibacteria genome, which is from an anaerobic bioreactor. It is 32.4% larger than the average size of all complete Saccharibacteria genomes (Table 1). By re-annotating and analyzing these previously reported complete genomes, we found that the *T*. *rhizospherense* genome encodes nearly 40% more unannotated genes as the other Saccharibacteria.

**Table 1:** Genome statistics for *T*. *rhizospherense* and other complete Saccharibacteria genomes

	Environment	Genome size (bp)	Gene number	Unannotated genes	Average gene size (bp)	Total coding sequence (bp)	Coding density (%)	CAZy genes	Unduplicated CAZy genes	Publication
Candidatus *T*. *rhizospherense*	Rhizosphere	1,450,269	1531	704	843.3	1,280,911	88	58	27	This study
47_87 scnpilot	Wastewater	900,471	918	376	896	822,552	91	30	17	[17]
49_20 scnpilot	Wastewater	904,897	933	362	891.5	831,756	92	28	14	[17]
GW2011 GWC2 44_17	Aquifer	1,038,683	1093	482	860.3	939,465	90	40	20	[1]
RAAC3_TM7	Aquifer	845,464	921	355	835.5	769,452	91	28	17	[8]
TM7x	Human mouth	705,138	711	241	919.2	653,574	93	30	17	[7]
Candidatus *S. aalborgensis*	Wastewater	1,013,781	1056	481	876.5	925,533	91	30	17	[16]
Difference from average		32.4%	33.2%	39.1%	− 3.7%	30.6%	− 3%	39.9%	31.7%	

There are 130 *T*. *rhizospherense* functional annotations that were not found in any other Saccharibacteria (152 genes have these annotations, with some annotated to have the same function). A few appear to be involved in amino acid metabolism, others in transcriptional regulation, DNA repair, sugar metabolism, transport, non-homologous end-joining, three genes annotated as NADPH:quinone reductase, and a dihydropteroate synthase for use in folate synthesis.

The genome appears to encode a nickel superoxide dismutase that may be used for oxidative stress response. The 49_20 scnpilot genome also encodes a gene with this function (but it is a Fe-Mn family superoxide dismutase). *T*. *rhizospherense* may have the ability to convert methylglyoxal to lactate, a two protein pathway (lactoylglutathione lyase and hydroxyacylglutathione hydrolase) that is absent in the other analyzed Saccharibacteria. This pathway is important in detoxification of methylglyoxal.

*T*. *rhizospherense* has a notably larger repertoire of carbohydrate active enzymes than occurs in the other Saccharibacteria, including 58 genes with 27 unique annotations. Included in the set and not found in other Saccharibacteria genomes are genes predicted to confer the ability to hydrolyze hemicellulose (AG-oligosaccharides and mannooligosaccharides), amino sugars (galactosaminide), and pectin (oligogalacturonides). The genome encodes a gene for transaldolase, a protein in the pentose phosphate pathway, which is absent in all other analyzed Saccharibacteria genomes.

*T*. *rhizospherense* is the only Saccharibacteria with a complete twin-arginine protein translocation system (*tatBC*). Four genes were predicted to have a TAT motif, two genes of unknown function, a phosphatidylglycerol lysyltransferase (discussed above and not present in any other analyzed genomes), and a MFS transporter, DHA2 family, methylenomycin A resistance protein.

Of the *T*. *rhizospherense* genes involved in phosphatidylinositol mannoside production, phosphatidylinositol alpha-mannosyltransferase is found only in *T*. *rhizospherense* and 47_87 scnpilot and alpha-1,6-mannosyltransferase is found only in Candidatus *T*. *rhizospherense* and Candidatus *S*. *aalborgensis*. None of the other genomes encode the plant hormone-related genes: “Lonely Guy” or salicylate hydroxylase.

The *T*. *rhizospherense* genome lacks several annotated genes found in most other Saccharibacteria. *T*. *rhizospherense* is unable to make (p)ppGpp, an alarmone that downregulates gene expression. Also lacking is dihydrolipoyl dehydrogenase, a subunit of pyruvate dehydrogenase (the function of which is unclear in these bacteria). Additionally not found are a *recJ* gene that encodes for a single-stranded DNA exonuclease, a glutamine amidotransferase involved in cobyric acid synthase, the *murC* gene involved in peptidoglycan synthesis, and the *pilW* gene involved in pilus stability.

## Discussion

The *T*. *rhizospherense* genome does not encode the capacity to generate the ribose backbone of DNA or bases and thus it must acquire nucleotides from external sources. Importantly, its DNA is labeled with ^13^C that likely originated from ^13^CO_2_ fixed by the plant over the 6-week study period. There are several sources of labeled DNA that might have been available to *T*. *rhizospherense*. We suspect that it obtained DNA from bacteria that lived off plant-derived carbon. However, some of the *T*. *rhizospherense* DNA might have been from phage that killed bacteria that grew on the ^13^C-labeled plant exudates. This is plausible because we identified labeled phage DNA (data not shown). Alternatively, *T*. *rhizospherense* may acquire DNA from fungi or the plant, but we consider this less likely given the predicted obligate association between Saccharibacteria and other bacteria.

As Saccharibacteria depend on extracellular nucleosides, they may have difficulty regulating the correct nucleoside concentrations. This is known to be a mutagenic condition and a possible reason for the large number of DNA repair mechanisms [44]. Further, multi-copy enzymes that prevent incorporation of damaged nucleotides may reflect dependence of externally derived DNA that may have been damaged during residence in the soil environment. The lifestyle of *T*. *rhizospherense* is novel compared to those of other CPR in that it is predicted to have the capacity to grow aerobically. Consistent with this, it has a number of oxidative stress response genes. A close relationship with plant roots also may explain the presence of these genes, as plants release reactive oxygen species in response to pathogens and stress. It is the first member of the Saccharibacteria phylum to be described from the rhizosphere, and it appears to be adapted to life there via an expanded genetic repertoire for plant hormone modulation and carbohydrate degradation relative to other Saccharibacteria.

It is possible that *T*. *rhizospherense* is a symbiont of soil-dwelling bacteria, with a lifestyle analogous to that of the Saccharibacteria TM7x that was cultured as an epibiont of a mouth-dwelling Actinobacteria [7, 18]. A similar association is indicated by the antibiotic resistance mechanisms that are encoded by *T*. *rhizospherense* that protect against compounds produced by Actinobacteria, methylenomycin A, oleandomycin, vancomycin, and streptomycin. Additionally, the predicted ability for *T*. *rhizospherense* to produce phosphatidylinositol mannosides provides an intriguing connection between this organism and the clades of Actinobacteria that produce phosphatidylinositol mannosides, *Mycobacterium* being the best studied. In *Mycobacterium*, this cell wall component may protect the cell from antibiotics and act as a virulence factor during infection [28].

We suspect that *T*. *rhizospherense* is a symbiont of a rhizosphere-dwelling organism. However, we do not know the extent of labeling, other than DNA, of cellular components of *T*. *rhizospherense.* These components may have derived from plant root exudates and would therefore be labeled, but we cannot say for certain as we did not measure the isotopic composition of lipids, proteins, and metabolites. The presence of machinery to degrade a variety of complex carbohydrates including cellulose and fungal cell walls suggests that this organism may not rely solely on plant exudates, but can also degrade biomass and necromass in the rhizosphere. *T*. *rhizospherense* has many genes for attachment that could be used to connect to a microbial host or plant surfaces. It may directly interact with the plant, given genes for the modulation of signaling molecules zeatin, cytokinin, and salicylic acid.

## Conclusions

To our knowledge, this is the first study to generate a complete, circularized genome from a soil-dwelling bacteria de novo from any soil metagenome. It is the first stable isotope-informed genome-resolved metagenomic study in the rhizosphere and thus the first soil microbiome study to make use of stable isotope probing to track carbon from atmospheric CO_2_ into the complete genome of a novel rhizosphere organism. The evidence indicates that ^13^C was incorporated into the *T*. *rhizospherense* DNA through its use of externally derived nucleic acids that may have been synthesized by associated organisms that grew on plant exudates. *T*. *rhizospherense* is the first genomically described rhizosphere-associated member of the Saccharibacteria phylum and many of its predicted metabolic capacities distinguish this organism from related Saccharibacteria that live in anaerobic environments. The rhizosphere soil may alternate between aerobic and anaerobic conditions, and it appears that *T*. *rhizospherense* may have adapted to these cycles by having the capacity to perform fermentation and to grow aerobically using an unusual electron transport chain.

## Methods

### Labeling

Soil (0–10 cm) was collected from the University of California Hopland Research and Extension Center (Hopland, CA, USA), from an area where *Avena* spp. are a common grass. Microcosms were constructed and plant growth conditions were regulated as described previously [45]. For this analysis, we used samples obtained from a single microcosm. A sample of T0 soil was collected after microcosm preparation and before planting. A single *A*. *fatua* plant was grown in a microcosm in a labeling chamber maintained at 400 μL/L CO_2_, with native CO_2_ replenished with 99 atom% ^13^CO_2_. After 6 weeks, a single sample of rhizosphere soil and a single sample of bulk soil was sampled at the vegetative stage; the microcosm was destructively harvested for rhizosphere soil and from bulk soil mesh bags which excluded root ingrowth. Rhizosphere soil was washed off the roots and DNA was extracted from 0.5 g of soil using a phenol:chloroform extraction detailed in [45]. We used bulk soil samples to control for the direct incorporation of ^13^CO_2_ into biomass. We used two different types of samples to control for non-plant-related ^13^CO_2_ influence: (1) DNA extracted from pre-planted microcosm soil which never received ^13^CO_2_ treatment and (2) DNA extracted from bulk soil collected at the same times as root harvesting (6 weeks) within the same microcosm [45]. The pre-planted sample provides a clean natural abundance bulk soil and the T6 bulk soil sample controls for any direct microbial carbon fixation. Plant isotopic composition was determined on an Isoprime 100 isotope ratio mass spectrometer (Elementar, Langenselbold, Germany).

### Stable isotope probing

To separate isotopically enriched DNA from unenriched DNA, each sample was separated based on density in a CsCl density gradient formed in an ultracentrifuge. Gradients were generated according to the method previously described [46]. Briefly, 5.5 μg of DNA was added to the gradient buffer to create a solution with a density of 1.735 g/mL. Then 5.2 mL of the solution was transferred to an ultracentrifuge tube (Beckman Coulter Quick-Seal, 13 × 51 mm). Tubes were spun in an Optima L-90K ultracentrifuge (Beckman Coulter, Brea, California, USA) using a VTi65.2 rotor at 44,000 rpm (176,284 RCF_avg_) at 20 °C for 109 h with maximum acceleration and braking of the rotor to maintain the integrity of the density separations. Then the content of the ultracentrifuge tube was separated into ~ 32 fractions using a syringe pump to deliver light mineral oil at 0.25 mL/min to displace the gradient solution from the pierced bottom of the tube. Each fraction was approximately 12 drops (~ 144 μL). The density of each fraction was measured using an AR200 digital refractometer (Reichert Inc., Depew, New York, USA). The DNA for each fraction was precipitated and quantified as previously described [46]. Fractions were then binned based on density and by comparison between the rhizosphere samples and the associated bulk soil (light = 1.692–1.737 g/mL; middle = 1.738–1.746 g/mL; heavy = 1.747–1.765 g/mL) (Additional file 5: Figure S3 and Additional file 1: Table S1).

### Sequencing

Each fraction, light, middle, and heavy, was then sent to the UC Davis Genome Center DNA Technologies Core for sequencing. Each sample was sequenced using an Illumina HiSeq 3000 (Illumina Inc., Hayward, California, USA) with paired-end libraries prepared with the Kapa Hyper protocol and a read length of 150 bp.

### Genome reconstruction, annotation, and analysis

Reads were trimmed using Sickle (https://github.com/najoshi/sickle); BBtools (https://sourceforge.net/projects/bbmap/) was used to remove Illumina adapters and trace contaminants; finally, reads were individually assembled using IDBA-UD (-step 20, -maxk 140, -mink 40) [47]. A single 1.45 Mb scaffold was recovered and was able to be circularized, then scaffolding errors and completeness were assessed as described in [1] and scaffolding gaps were fixed manually by mapping reads to the scaffold using Bowtie2 on default settings [48]. The scaffold was visualized in Geneious [49]. Genes were predicted using Prodigal [50]. Predicted ORFs were functionally described using a multidatabase search pipeline. Sequence similarity searches were performed with USEARCH [51] against UniRef100 [52], Uniprot [53], and the KEGG database [54]. Additional gene annotations were assigned using HMMs that were constructed based on KEGG Orthologies [54]. All the proteins assigned to a KO were clustered using MCL [55] with inflation parameter (-I) of 1.1, based on global percent identity. Clusters were aligned using MAFFT v7 [56], and HMMs were constructed using the HMMER suite [57]. Carbohydrate-active enzymes were identified using dbCAN [58]. Domain level functional annotations were done using InterProScan [59]. tRNAs were predicted using tRNAScan-SE [60], and cellular localization was predicted using PSORTb v3.0.2 with the gram-positive setting, which was demonstrated though previous imaging [4, 61]. Twin-arginine translocation signal peptide finding was done using TATFIND 1.4 [62]. The GC skew of the genome was calculated based on previously published tools [63]. Protein modeling was done with Swiss Model, Ndh modeling incorporated the protein from *Caldalkalibacillus thermarum*, and cytochrome bo_3_ ubiquinol terminal oxidase subunit I utilized a protein model from *E*. *coli* [33, 34, 64]. Representative Saccharibacteria 16S rRNA sequences were obtained from NCBI and aligned using ssu-align [65], then a maximum-likelihood tree was constructed with RAxML by using the GTRCAT model with 1000 bootstraps (Additional file 6: Figure S4).

## Additional files

Additional file 1: **Table S1.** Stable isotope probing and sequencing statistics. (XLSX 9 kb)

Additional file 2: **Table S2.** Genome summary, including HMM-based annotations. (XLSX 1076 kb)

Additional file 3: **Figure S1.** Plot of GC skew (black) and cumulative GC skew (green, window 1000 bp, slide of 10 bp) of the *T*. *rhizospherense* genome. The plot shows the predicted locations of the origin (red line, 1201 bp) and terminus (blue line, 757,370 bp) of replication. The form of the plot is as expected for a correctly assembled, circularized genome that undergoes bi-directional replication from a single origin. (PDF 69 kb)

Additional file 4: **Figure S2.** Protein modeling of *T*. *rhizospherense.* Proteins with Swiss-Model in Clustal colors with DSSP secondary structure overlaid. **a** The top row is the *T*. *rhizospherense* NADH dehydrogenase II and the bottom row is the reference protein from *Caldalkalibacillus thermarum.* The symbols above the alignment indicate: *F* FAD binding site, *N* NADH binding site, and *U* Ubiquinone or menaquinone binding site. **b** The top row represents the *T*. *rhizospherense* cytochrome bo_3_ ubiquinol oxidase subunit I and the bottom row is the reference sequence from *E*. *coli*. The symbols above the alignment represent key residues: *D* D-channel, *U* Ubiquinol binding site, *. Ion binding site, *K* K-channel, bulky hydrophobic residues which differentiate between cytochrome c oxidase and cytochrome bo_3_ ubiquinol oxidase. (PNG 3927 kb)

Additional file 5: **Figure S3.** The same tree as in Fig. 2 with accessions. (PDF 93 kb)

Additional file 6: **Figure S4.** Stable isotope fraction determination from Fig. 1 with added pre-planted bulk soil. (PDF 283 kb)
