# Supplementary figures and images for: Stable isotope informed genome-resolved metagenomics reveals that Saccharibacteria utilize microbially-processed plant-derived carbon
Source: Microbiome. 2018 Jul 3;6:122. doi: 10.1186/s40168-018-0499-z (PMC6031116; doi:10.1186/s40168-018-0499-z)

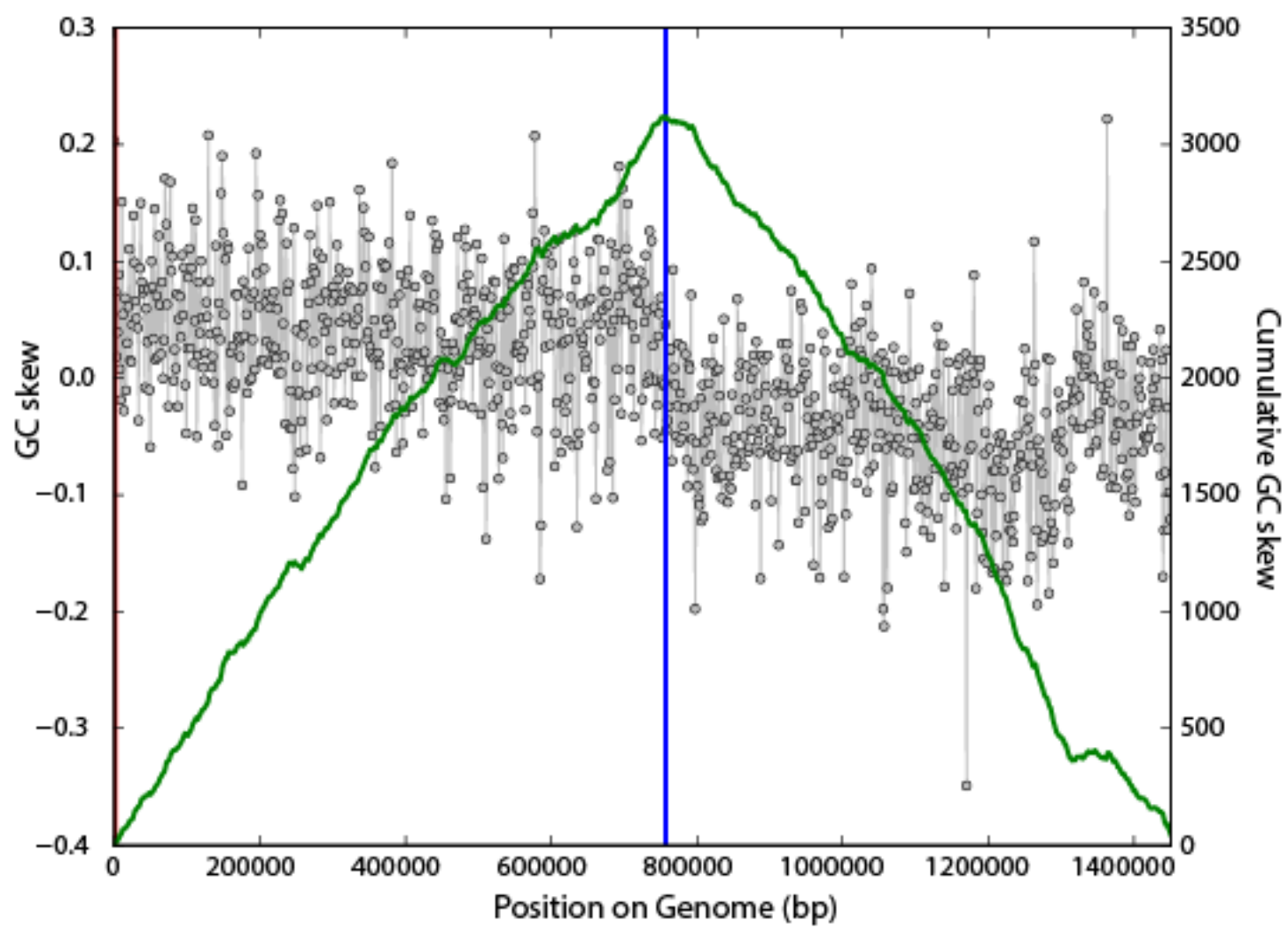

Supplement: Supplementary file 3 — Figure S1. Plot of GC skew (black) and cumulative GC skew (green, window 1000 bp, slide of 10 bp) of the T. rhizospherense genome. The plot shows the predicted locations of the origin (red line, 1201 bp) and terminus (blue line, 757,370 bp) of replication. The form of the plot is as expected for a correctly assembled, circularized genome that undergoes bi-directional replication from a single origin. (PDF 69 kb) [file 40168_2018_499_MOESM3_ESM.pdf]

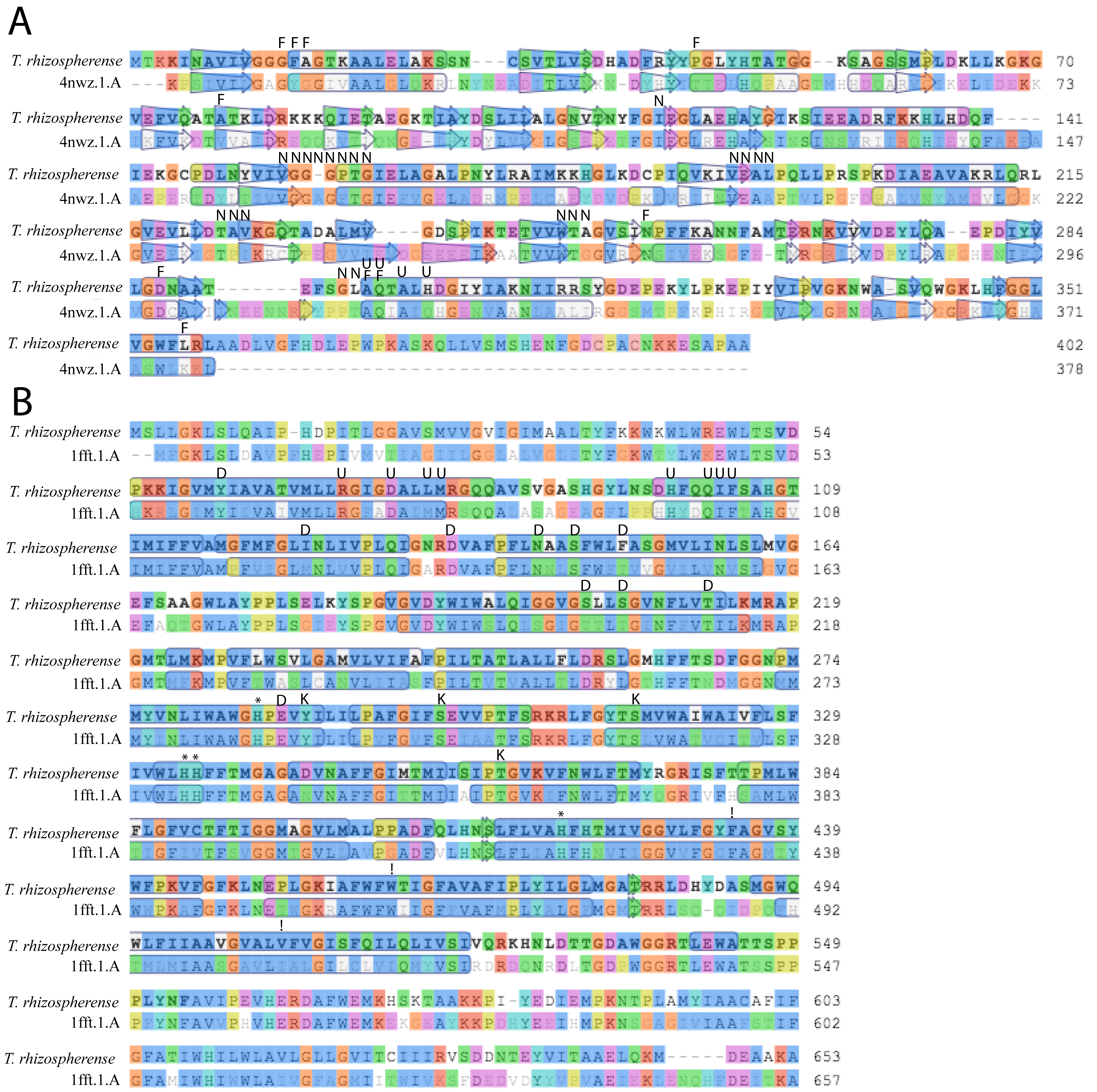

Supplement: Supplementary file 4 — Figure S2. Protein modeling of T. rhizospherense. Proteins with Swiss-Model in Clustal colors with DSSP secondary structure overlaid. a The top row is the T. rhizospherense NADH dehydrogenase II and the bottom row is the reference protein from Caldalkalibacillus thermarum. The symbols above the alignment indicate: F FAD binding site, N NADH binding site, and U Ubiquinone or menaquinone binding site. b The top row represents the T. rhizospherense cytochrome bo3 ubiquinol oxidase subunit I and the bottom row is the reference sequence from E. coli. The symbols above the alignment represent key residues: D D-channel, U Ubiquinol binding site, *. Ion binding site, K K-channel, bulky hydrophobic residues which differentiate between cytochrome c oxidase and cytochrome bo3 ubiquinol oxidase. (PNG 3927 kb) [file 40168_2018_499_MOESM4_ESM.png]

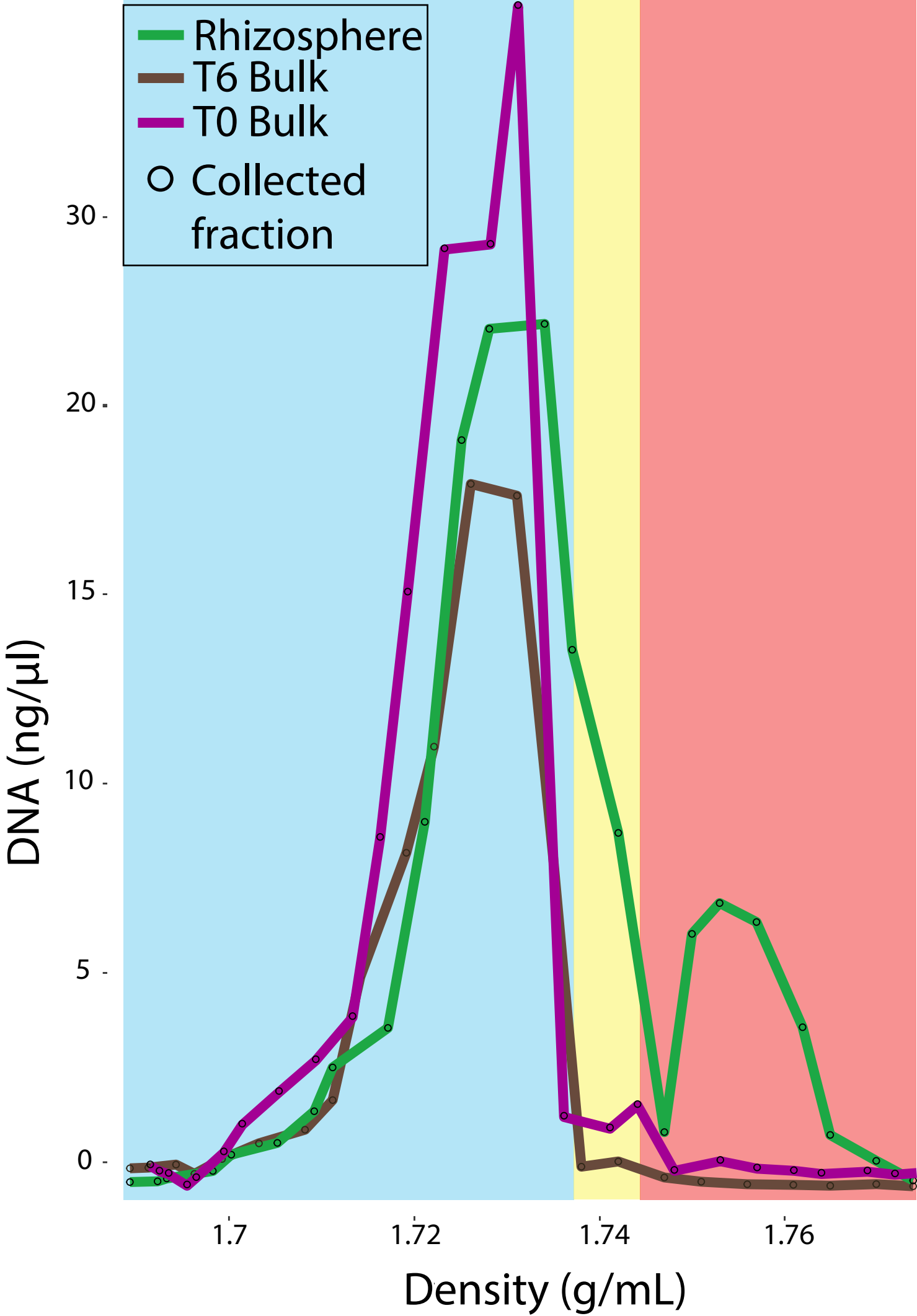

Supplement: Supplementary file 5 — Figure S3. The same tree as in Fig. 2 with accessions. (PDF 93 kb) [file 40168_2018_499_MOESM5_ESM.pdf]

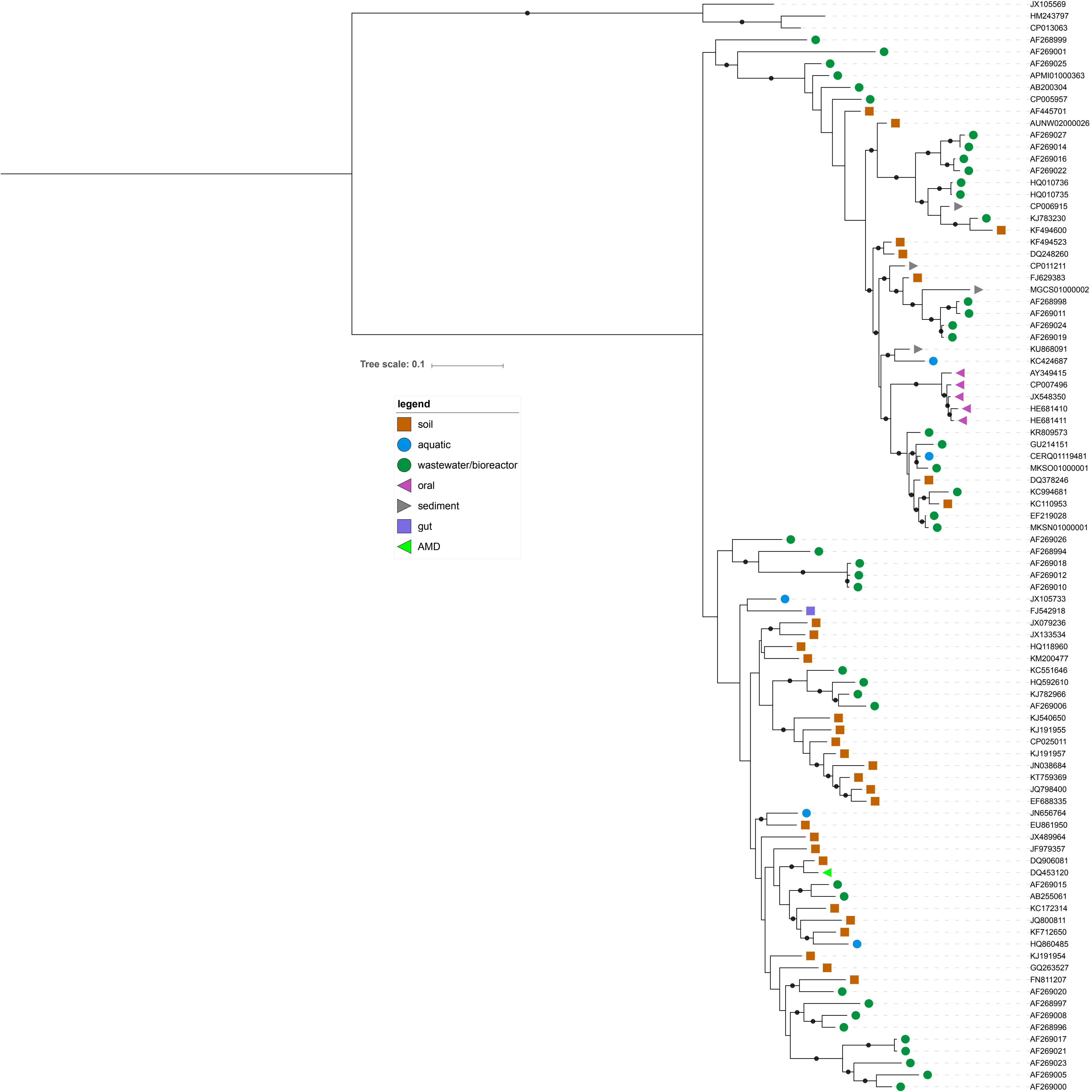

Supplement: Supplementary file 6 — Figure S4. Stable isotope fraction determination from Fig. 1 with added pre-planted bulk soil. (PDF 283 kb) [file 40168_2018_499_MOESM6_ESM.pdf]
